# Application of space-time disease clustering by administrative databases in Italy: Adverse Reproductive Outcomes (AROs) and residential exposure

**DOI:** 10.1186/s12963-015-0070-0

**Published:** 2015-12-24

**Authors:** Pamela Barbadoro, Massimo Agostini, Marcello M. D’Errico, Francesco Di Stanislao, Fabio Filippetti, Sara Giuliani, Emilia Prospero

**Affiliations:** Department of Biomedical Science and Public Health, Università Politecnica delle Marche, Via Tronto 10/a, Ancona, 60125 AN Italy; Local Health Authority, ASUR Area Vasta 1, Fano, Italy; Regional Epidemiology Network, Ancona, Italy; School of Hygiene and Preventive Medicine, Università Politecnica delle Marche, Ancona, Italy

**Keywords:** Cluster analysis, Environmental exposure, Health information systems, Pregnancy outcome, Small area, Italy

## Abstract

**Background:**

The aims of this study were to estimate the existence of clusters of AROs in the municipalities of the Marches Region (Central Italy) after complaints from residents living near an abandoned landfill site.

**Methods:**

Cases of AROs (i.e., congenital malformation, chromosomal abnormalities, and low birth weight) were retrieved from hospital discharge data. SaTScan and GeoDa were used to check for the presence of clusters at a regional and a small area level. Moreover, at a small area/neighborhood level, smoothed rates were calculated, and a case–control approach was used to assess the residence in proximity to the abandoned landfill as an independent risk factor for AROs.

**Results:**

AROs were associated with the price per square meter of the accommodations in the area of residence (OR 2.53, 95 % CI 2.06-3.10). On the other hand, residence within one kilometer of the landfill (OR 0.04, 95 % CI 0.01-0.23) and maternal age greater than 35 years (OR 0.96, 95 % CI 0.92-0.99) were protective.

**Conclusions:**

Residency in proximity to the abandoned landfill was not a risk factor for the occurrence of AROs. The results show that basic information, such as the price of accommodations in different neighborhoods, could be of interest in order to target training programs for women living in difficult conditions and highlights the potential role of the building environment in perinatal health. However, we note that aside from the data provided by Geographic Information Systems in public health, collection of the patient’s residential address was unreliable for selected conditions. Future efforts should emphasize the patient’s residential address as information important for evaluating the health of individuals instead of being merely administrative data.

## Introduction

In autumn 2006, the population living in Fano (a town located on the Adriatic coast of the Marches Region in Italy) began to complain about a presumed high frequency of congenital malformations and other adverse birth defects in an area surrounding an abandoned landfill site. As a consequence of these complaints, a cluster investigation was carried out. The aims of this study included regional and small area approaches. For the regional approach, the aims were to estimate the Adverse Reproductive Outcomes (ARO) incidence rate at a municipality level and to evaluate the existence of clustering of AROs in the municipalities of region. At a small area level, in the town of Fano, the incidence of AROs was evaluated by neighborhood, a cluster analysis was performed, and the proximity of the residence to the landfill site was assessed as an independent risk factor for ARO occurrence.

The analysis of epidemiologic data at a small area level has been increasingly used to measure the need for target interventions and to evaluate the impact of local health policies [1]. Small-area studies investigate the role of the neighborhood level in population health, and most of them have found detrimental health impacts on residents of deprived neighborhoods [2, 3]. The specific value of small-area analysis is that it permits the examination of data for populations that tend to be more homogeneous in character and environmental circumstances than the larger and more widely spread populations [3]. In fact, besides the great capability of these studies to integrate information from multiple levels of interest (social, personal, etc.), these methods are also interesting for assessing human exposure to environmental pollution, especially in large studies when an approach based on individual exposure is often rather demanding for participants and requires extensive, thus often inadequate, resources. Therefore, the need to analyze a spatial gradient in environmental epidemiology is crucial. Multiple approaches have been used to estimate exposure of individuals, using the area of residence as a proxy [4, 5]. The smallest territorial unit that can be used in small area studies depends on data availability that may vary in different countries. For example, in Italy small area studies can be carried out at the census tract (average of 200 residents) and municipality levels [6].

Administrative databases represent a useful source of information to study the epidemiology of diseases and to analyze trends of various health conditions [7]. They offer important benefits from a practical point of view, and in recent years they have become a basic source of data for disease surveillance, evaluation of health resource use, and assessment of healthcare outcomes [8, 9]. Recently, many studies have explored spatio-temporal patterns of disease incidence in order to identify areas of significantly elevated or decreased risk and to suggest potential causes [10, 11]. An exploration of the spatio-temporal clustering of the incidence of a given disease can provide useful information for policymakers and health planners in their studies of possible ecological or group-level risk factors and in focusing the administration of specific public health initiatives [11].

Among the major concerns of public health importance is the possible impact of environmental pollutants on the developing fetus. The linkage between built environment and socioeconomic conditions has been increasingly identified in recent years [12], and the effects of the built environment on perinatal health at the neighborhood level may be mediated by different mechanisms [13–16]. In this context, it has been reported that the linkage between characteristics of the built environment of mothers and their socioeconomic position may partially confound some of the long-observed association between exposure to environmental factors and adverse health outcomes [17–19].

## Methods

### Study area

**Fig. 1:**
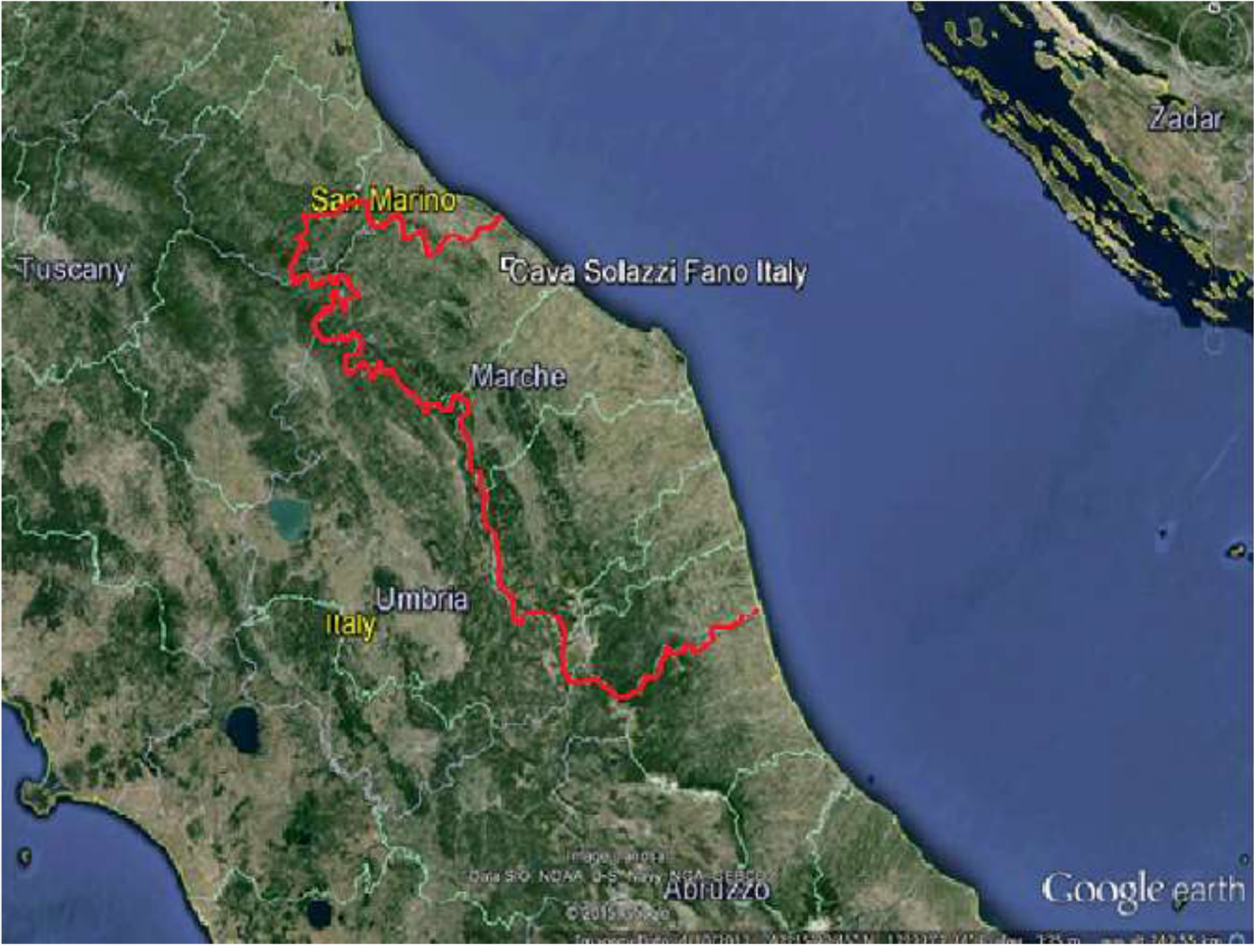
The Marches region in Italy

The Marche region is located on the Adriatic Sea, in Central Italy, and the town of Fano is located in the northern part of it (Fig. 1). The region had a total population of 1,450,000 inhabitants at the time of the events, while the town of Fano had about 40,000 inhabitants. Soil samples from the abandoned landfill site were analyzed by the local Environmental Health Authority, which did not find any particular contamination by known toxic substances. The landfill site had been in use until 2003 and then abandoned.

### Case definition

Since the Marches Region did not have a congenital malformations (CM) registry at the time of the events [20], the integration of different existing healthcare information systems was used to evaluate the phenomenon. An Adverse Reproductive Outcome (ARO)-associated hospitalization was defined as hospitalization occurring at birth, or during the first year of life, for which one of the ICD-9-CM codes for CM or low birth weight (LBW) was listed in any of the discharge diagnosis fields. AROs were analyzed as a whole and according to the following classes: malformations of the central nervous system, cardiovascular malformations, orofacial malformations, ear malformations, gastrointestinal malformations, genitourinary malformations, and musculoskeletal malformations. Infants with more than one malformation were counted in each relevant category; individual cases were traced through the analysis of hospital discharge records (HDR), and repeated hospitalizations were excluded from the analysis. The one-year term of observation from birth was used to enhance the sensitivity of case identification. The analysis of HDR was also used to detect cases of voluntary termination of pregnancy (ICD-9 635) with a secondary diagnosis of malformation of the CNS (ICD-9 655.1), as they were likely to be due to cases of CM identified through prenatal diagnosis. Similarly, cases related to the prenatal diagnosis of chromosomal abnormalities (CA) were identified and included in the ARO definition.

### Regional-level analysis

The first step of this analysis was done by carrying out a retrospective cohort study in order to assess the risk of AROs in the resident population of the town compared with that of the resident population in the same region.

Rates were calculated by use of denominators derived from the Italian National Institute of Statistics (http://demo.istat.it/) and were expressed as the estimated number of cases per 1,000 infants. According to the EUROCAT methodology [21] for calculation of rates, a newborn with several anomalies is counted once within each class of anomaly. Therefore, the number of cases in different classes cannot be added to reach a total number of single cases. A baby is counted once in the calculation of ARO rates, even if affected by multiple AROs. Confidence intervals were calculated by using Poisson approximation. A multilevel mixed-effects linear regression modeling approach was used to evaluate the variables related to AROs. The model included local health authority, the period of study, mean age of mothers at delivery, and deprivation index by municipality.

The model design followed the general format of generalized linear mixed effects models; in particular, the AROs incidence rate at the regional level (y_*ij*_) was assumed to be associated with two level-related factors—municipality level and local health authority level, as follows:

$$ {}_{yij}={\upbeta}_{0\mathrm{j}}+{\upbeta}_1{\mathrm{x}}_{1\mathrm{i}\mathrm{j}}+{\upbeta}_2{\mathrm{x}}_{2\mathrm{i}\mathrm{j}}+{\upbeta}_3{\mathrm{x}}_{3\mathrm{i}\mathrm{j}} + {\mathrm{u}}_0\mathrm{j}+{\mathrm{e}}_{\mathrm{ij}} $$

In this model, three fixed coefficients at the municipality level (_i_), the mean age of mothers, year of events, and the deprivation index have been introduced, while as a second-level factor (_*j*_), the local health authority of residence has been considered.

Among the possible confounding variables available through the current health information system, mean maternal age at delivery at a municipality level and an index of local deprivation were selected for evaluation. The deprivation index included in the regional analysis at a municipality level was calculated according to the index validated by Cadum and included the following variables: percentage of single-parent families, unemployment rate, percentage of people with a primary education, the percentage of homes without a bathroom in the house, and the percentage of households that rent [22].

The SaTScan™ software was used for analysis of clusters of AROs in the different municipalities of the region [22, 23]. SaTScan statistic identifies the most likely (unusual) cluster [24–27]. A Poisson model was used during the analysis. Clustering was performed using purely spatial, temporal, and spatial-temporal scenarios, separately. The maximum cluster size was set at different levels (i.e., 20 % and 50 % of the total population at risk), and for temporal analysis a one-year interval was chosen. For the analysis of clusters, distribution of cases by municipality was assessed; the center of each of the 246 towns belonging to the Marches Region was calculated, and their coordinates have been used as reference positions for each geographic entity.

Although the spatial scan statistic used in SaTScan is widely accepted, there are acknowledged sensitivities of results depending on input parameters. For instance, Tango [28] pointed out that when using SaTScan, often the most likely cluster is very large and “swallows” neighboring regions that have non-elevated risk. In conjunction with SaTScan analysis, cluster detection was carried out with the help of the GeoDa software, calculation of smoothed rates, and computation of Local Moran’s I [29]. Moreover, local indicators of spatial association (LISA) maps generated by a Local Moran statistical test were used to visualize clusters. Significance of clusters was assessed using Monte Carlo simulations with 999 permutations.

### Small area-level analysis, Fano municipality

Through the linkage of data provided by the georeferencing service active in the municipality of interest, the census office of the same municipality, and the data processing center of the local health authority, it was possible to georeference individual cases of AROs occurring in Fano. The cases and controls for which it was not possible to associate a single address at the time of pregnancy and delivery were excluded from subsequent analysis. Moreover the municipality’s georeferencing service has provided the exact coordinates of location of the former landfill site. In particular, HDR and personnel data were linked by the local health authority’s data-processing center to obtain the full names of newborns. Personnel from the data-processing center did not know the study’s objective or the health status of newborns. The names, ages, and addresses of mothers at the time of delivery were ascertained through the linkage of data provided by Fano’s census office. With the help of the georeferencing service active in the same municipality, it was possible to georeference individual cases of AROs occurring in the town. Cases of AROs were then grouped at a neighborhood level, and rates underwent Bayesian smoothing by the GeoDa software. LISA maps generated by a Local Moran statistical test were used to visualize clusters; significance of clusters was assessed using Monte Carlo simulations with 999 permutations. Cluster analysis was performed by SaTScan both on data aggregated by electoral arrangement and by means of a case control approach, using newborns’ addresses at birth. The maximum cluster size was set at different levels (20 % and 50 % of the total population at risk), and for temporal analysis a one-year interval was chosen.

Finally, a case–control study was carried out to assess the risk of AROs in infants born in the index town during the period 2001 to 2006. At least two controls were initially selected for each case from hospital discharge data, with a random sample stratified by year of birth. After record linkage with municipality records, exact address at the time of birth was available in 97.5 % of cases and 62.4 % of potential controls previously identified on the basis of hospital discharge data. The final number of controls was 331, with a ratio of 1.6 controls for each case.

Cases and controls were stratified by risk variables such as proximity to the former landfill site, maternal age at birth greater than 35 years, presence of comorbidities during pregnancy (i.e., diabetes, mood disorders requiring hospitalization, alcohol abuse, and use of toxic substances requiring hospitalization), and the value of the house of residence. The latter was classified into five levels according to the assessed price per square meter of the accommodations in the area of residence from the Italian Revenue Agency [30]. The incidence of ARO for each electoral arrangement of the town was calculated, together with the incidence of the CMs by *apparatus*, in toto, and LWB.

Multilevel logistic regression models were developed to adjust for confounding and to evaluate which factors were independently associated with an ARO. The criteria for entry of the variables in the model were selected from among those with a value lower than 0.20 in the bivariate, using the stepwise method. Independent variables were coded as follows: residence in the vicinity of the former landfill site (more than three kilometers (km) = 1; residence less than one km = 2, between one and two km = 3, between two and three km = 4), maternal age of 35 years or more at the time of delivery (yes = 1, no = 0), and class of price per square meter of the accommodations in the area of residence (categorized into quintiles: 1 = higher, up to 5 = lower). The significance level was set at *p* < 0.05.

## Results

### Regional-level analysis

Between 2001 and 2006, 77,812 infants under 1 year of age were identified by the Hospital Discharge Database (HDD) in the towns of the Marche Region. An ARO was detected within the first year of life for 5,871 of them, with an incidence rate of 75.45 per 1,000 infants (95 % CI 73.60-77.33 per 1,000).

**Fig. 2:**
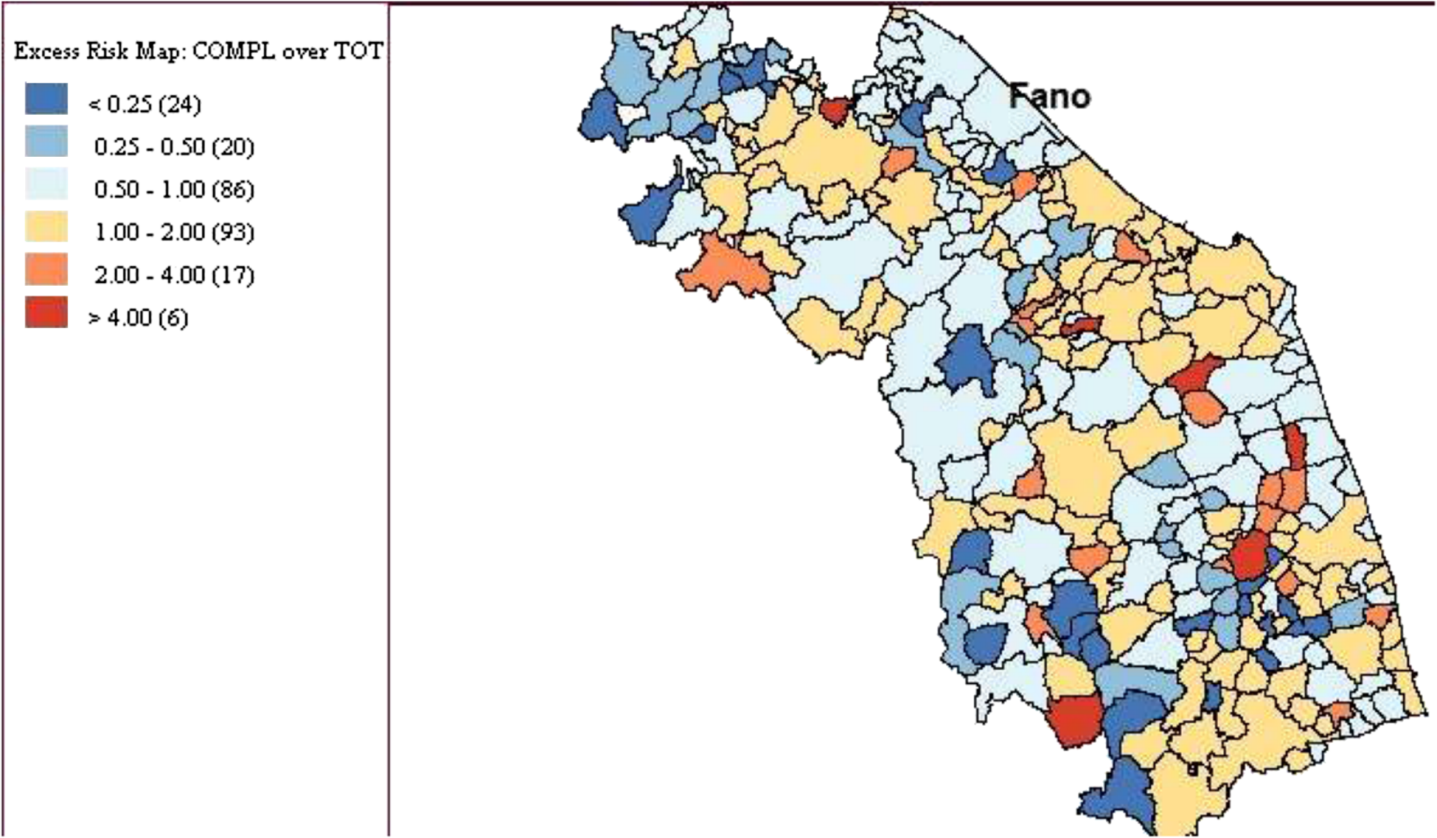
Distribution of Bayesian smoothed ARO rates in the Marches region

**Fig. 3:**
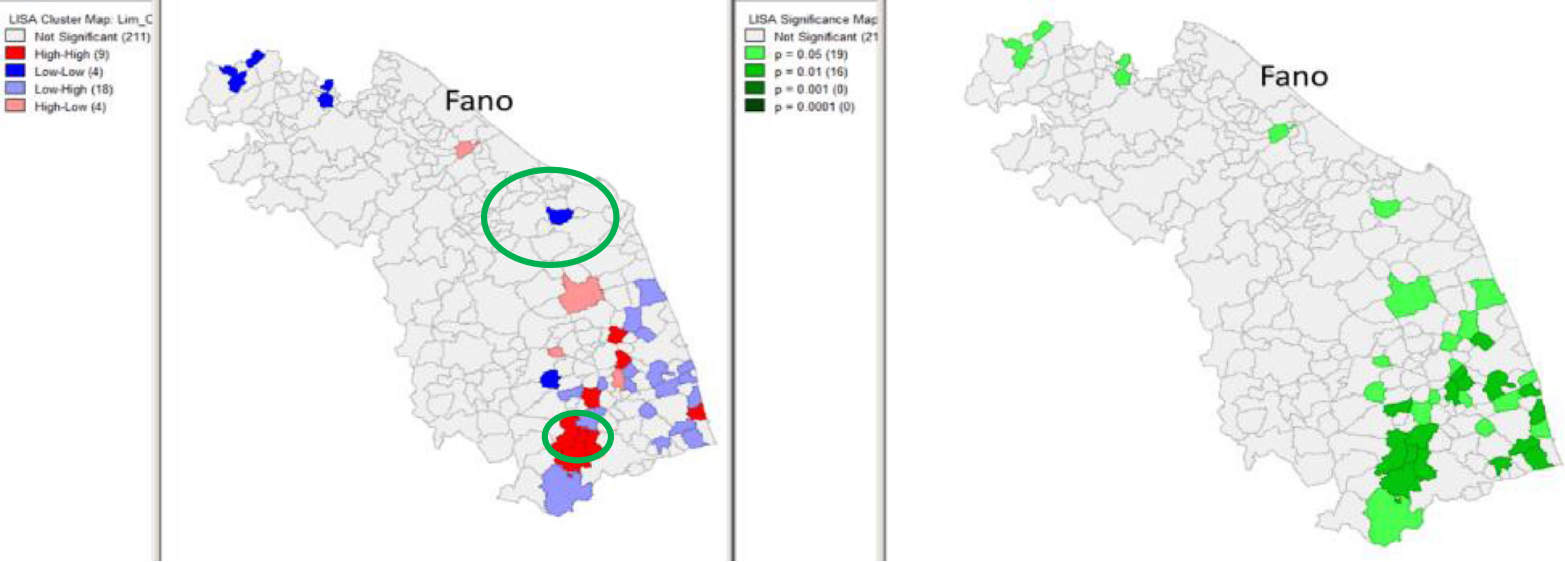
Distribution of most likely SaTScan clusters (green circle), and LISA cluster map and cluster significance (999 permutations) of AROs, Marches, 2001 to 2006

In Fano, the incidence rate of newborns with AROs was lower than that registered in the other municipalities of the region (with 213 AROs detected in 3,409 infants, an incidence rate of 62.48 per 1,000 infants, 95 % CI 54.59-71.13 per 1,000). In particular, while the incidence of CM and CA was similar to that registered in the whole region, LBW was less common in the index city. The detailing of cases by type of ARO is reported in Table 1. Distribution of Bayesian-smoothed ARO rates in the Marches Region is represented in Fig. 2. In particular, the SaTScan procedure, whether using the 20 % of the total population at risk or the 50 %, identified a most-likely cluster that included 19 municipalities (the cluster radius was 14.92 km, RR 1.68, *p* < 0.001). The space-time permutation found the same cluster in the 2003 to 2005 period, while the temporal approach found a not-significant cluster in the 2002 to 2004 period. Moreover, four secondary clusters have been identified (Fig. 3). The Local Moran’s I, represented by the LISA map, highlighted different municipalities showing high rates (Fig. 3). However, none of these clusters involved the town of Fano.

**Table 1:** Distribution of cases of CMs, chromosomal abnormalities, and low birth weight rates in the city of Insistence (of the landfill site) and in the Marches Region, 2001 to 2006

	Fano			Marches			∆
	*n*	Rate per 1,000	95 % CI	*n*	Rate per 1,000	95 % CI	
Congenital malformations							
Central nervous system	-	-	-	23	0.30	0.18–0.44	-
Cardiovascular	34	9.96	6.89–13.91	980	12.59	11.79–13.43	-
Orofacial	6	1.76	0.64–3.82	125	1.94	1.33–1.91	-
Ear	3	0.88	0–5.84	25	0.32	0.07–0.71	-
Gastrointestinal	4	1.17	0.31–2.99	114	1.77	1.2–1.75	-
Genitourinary	17	4.98	2.89–7.97	543	6.98	6.49–7.52	-
Musculoskeletal	24	7.03	4.5–10.45	583	7.51	6.89–8.13	-
Total	122	35.72	29.66–42.65	3375	43.65	41.88–44.86	-
Chromosomal anomalies	9	2.64	1.14–4.76	86	1.11	0.88–1.36	-
Low birth weight	82	24.05	19.38–29.70	2623	33.71	32.46–35.00	*
Total number of AROs^a^	301	88.30	78.98–98.32	7894	75.45	73.86–77.07	*

*p < 0.05

^a^Please see the Methods section for calculations used

The multilevel mixed-effect linear regression model showed that ARO rates were related to deprivation index in the area of residence and were more frequent in the second period of observation; mean mothers’ age at delivery was not independently related to the occurrence of AROs (Table 2).

**Table 2:** Regression coefficients of model covariates for estimating ARO rates at a municipality level

Effect	Coefficient	SE	*P* value
Mean mothers’ age	0.0002	0.0003	−0.0002–0.0008
Deprivation index	0.0022	0.0002	0.0018–0.0025
Period (2004 to 2006)	0.0007	0.0003	0.0001–0.0013
Variable	0. 046	0.009	0.03–0.065

### Small area study

**Fig. 4:**
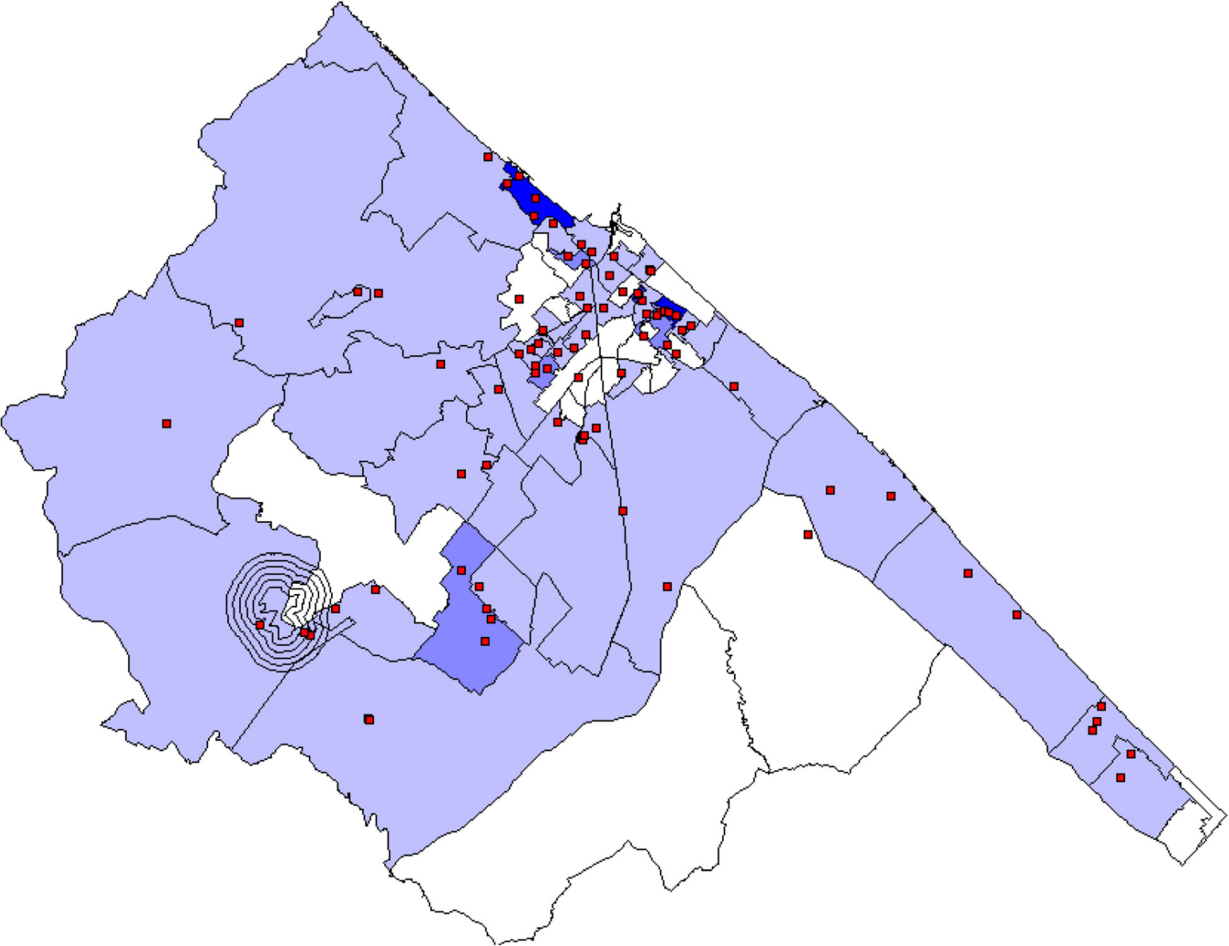
Colorimetric map showing the distribution of raw ARO rates in the different Fano neighborhoods, the distribution of cases (red points), and the position of the abandoned landfill site, with perimeter and 200-meter distance limits

**Fig. 5:**
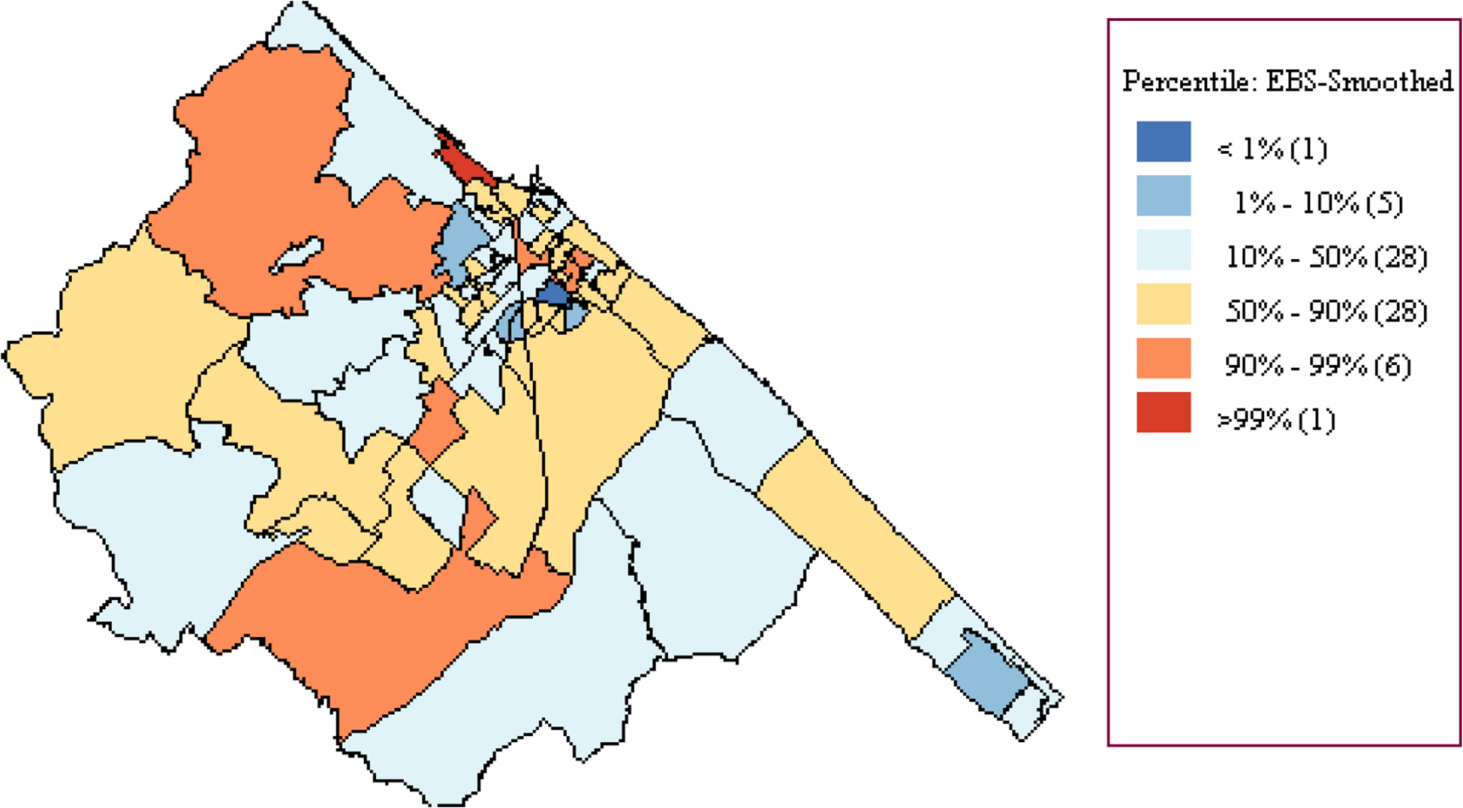
Map of the distribution of smoothed ARO rates in the different Fano neighborhoods

**Fig. 6:**
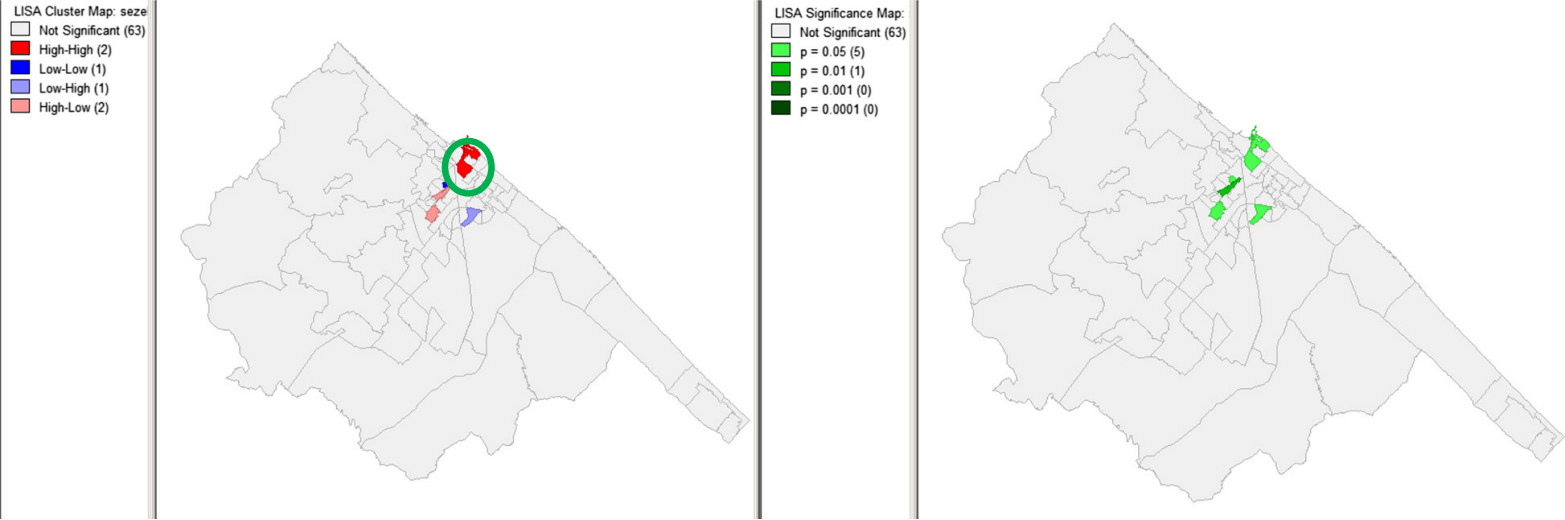
Distribution of most likely SaTScan clusters (green circle), and LISA cluster map and cluster significance (999 permutations) of AROs in Fano, 2001 to 2006

Rates calculated at the census tract level are represented in Fig. 4 (raw) and Fig. 5 (after Bayesian smoothing with the GeoDa software). Moreover, Fig. 4 details the georeferenced cases and controls, as well as the position of the abandoned landfill site. Cluster analysis, performed by SaTScan both on data aggregated by electoral arrangement and by using the coordinates of residence available for cases and controls, allowed the identification of a single cluster of AROs at a distance of about six kilometers from the landfill site in the 2001 to 2003 period. The cluster was also detected by the use of Local Moran’s I statistic, and visualized by the Moran’s I and LISA (Fig. 6).

The final sample for the small area study included 207 cases and 331 controls. The study of variables associated with congenital malformations has highlighted the role of price per square meter of the accommodation of newborns (OR 2.53, 95 % CI 2.06-3.10) (Table 3). Conversely, increase in maternal age was protective (OR 0.96, 95 % CI 0.92-0.99).

**Table 3:** Factors associated with AROs at multivariate analysis, *p* <0.001

	OR	95 % CI	*p*
Proximity to former landfill site			
Residence over 3 km from the site	1		
Residence 2–3 km from site	0.28	0.14–0.59	0.001
Residence 1–2 km from the site	0.61	0.18–2.03	0.419
Residence <1 km from the site	0.04	0.01–0.23	0.000
Low price per square meter of accommodations	2.53	2.06–3.10	0.000
Mother’s age >35 years	0.96	0.92–0.99	0.046

Residence within one kilometer of the landfill reduced the risk of AROs by 96 % (OR 0.04, 95 % CI 0.01-0.23); although not significant, the gradient of risk seems to move away from the former landfill site to increase beyond three kilometers.

## Discussion

The results reveal a lack of association between living near the former landfill site and any ARO. The finding of a reduced risk of ARO in proximity to the landfill site may seem in contrast with the general hypothesis linking environmental exposure to fetal adverse events. However, we must underline the absence of any known toxic substance from the landfill in this case. Moreover, other evidence from the literature has similarly found mixed [31] or negative [32] results.

Both the analysis performed at a regional level and that performed at a small area level highlight that the only independent factor significantly associated with an adverse outcome was a low price per square meter of the accommodations in the area of residence.

If we consider the low price per square meter of houses as a good proxy of the socioeconomic position of individuals, the above results are in agreement with those by Vrijheid and colleagues, highlighting an increased risk of congenital malformations at increasing social deprivation [33]. Timmermans suggests that among women who live in situations of distress, several variables that adversely affect the products of conception (such as smoking, abuse of alcohol and drugs during pregnancy, poor eating habits, lack of use of multivitamin supplements, and lack of interest in health and hygiene standards in general) may be simultaneously present [17]. On the other hand, older mothers seem to be protected from AROs, probably because of the availability of free prenatal screening. Moreover, the above studies have been confirmed by a recent meta-analysis concluding the detrimental role deprived areas have on perinatal outcomes [18]. Our data, although based on a limited number of observations, suggest that the people who live in conditions of socioeconomic disadvantage may have a higher risk of giving birth to infants with congenital malformations. These results confirm the association between poor neighborhoods and adverse pregnancy outcomes, and, in particular, that a meaningful relationship between the quality of the residential built environment and birth outcomes may be of interest as a good measure of general community health [2, 6, 12, 34, 35].

A wide range of maternal, socioeconomic, and environmental factors may mediate the impact of socioeconomic status on the prevalence of congenital anomalies, including personal factors (i.e., nutritional factors, parity and maternal age, maternal distress, and ethnic origin), as well as lifestyle factors (e.g., smoking habits), environmental and occupational exposures, and access to and use of health care services [13, 36–40]. In this context, information bias has been highlighted as one of the main findings of the paper; given a multifactorial condition, such as the AROs, the availability of a pathology registry is of crucial importance. We may discuss our results in light of a general lack of clusters; however, we were not able to inspect some well-known risk factors for congenital anomalies at an individual level. Spatial clustering maintains its importance in order to highlight possible foci; however, its integration with personal, socioeconomic, organizational, and political aspects is crucial, especially when dealing with complex events.

Moreover, when analyzing individual outcomes at an area level we should not forget the ecological fallacy, which links correlations observed at the group level to individuals. Ecological studies can provide useful exploratory information, but conclusions about individuals may be only weakly supported by data on groups.

In light of those limitations, we think the most important result of the study was the lack of availability of reliable health care data, affecting the ability to correctly verify the existence of clusters and the possible association with the various risk factors using the current information systems. In fact, data collection was carried out by a complex linkage between hospital, administrative, and geospatial data, rather than of pathology registries, leading to a loss of information both quantitative (it was possible to retrieve more specific data at an individual level, such as address of residence at the time of the events, mother’s name, for only 59 % of cases and 44 % of controls selected from the regional database) and qualitative (lack of personal risk factors). Intrinsic and relevant are the limits to the use of data relating to hospital admissions linked to the coding of diagnoses that may be relatively unreliable for reasons of expediency of the dispensers, for the inevitable errors of accuracy, precision, and reproducibility encoding the clinical data.

Moreover, we think that the above errors may even be more frequent when dealing with AROs, since we have had the need to link health data belonging to two different persons, the mother and the newborn, usually having a different family name and sometimes a different municipality of residence. These difficulties are even more important when reviewing rare events over time since the retrieval of information about the actual address at the time of events may be challenging. We should not forget the important role played by small numbers when dealing with rare events in small population [41].

Despite indicating some feasibility of cluster analysis at a municipality level by the current health information system, the lack of a congenital anomalies registry is of crucial importance in adequately assessing confounding factors. This puts into evidence, once more, the need for careful design and analysis to better characterize environmental effects on human reproduction [42]. Registries are one of the most accurate instruments for case ascertainment and surveillance of health events, assuring reliability in estimating rates of AROs [43]. Nevertheless, limited resources in birth defect surveillance programs sometimes require the use of electronic health archives–already available and designed for administrative purposes–for surveillance by public health researchers [44, 45]. In this context, hospital discharge data are increasingly used to estimate the occurrence of a wide range of diseases and, more recently, to estimate neonatal morbidity and birth defects at a national level in Italy [46].

These considerations point out the need for effective and up-to-date surveillance systems as active companions in monitoring healthcare phenomena. Aside from the ongoing advancement and crucial importance of utilizing Geographic Information Systems in public health, at least in this region, the routine activities of collecting the residential address of the patient seems to be a bit outdated and unreliable. Also, with respect to cancer epidemiology, the challenges associated with successfully identifying community clusters of disease is a real current problem [47]. Future efforts may be placed on emphasizing the importance of residential address as information strictly associated with the health of individuals instead of being merely administrative data. On the other hand, the importance of the accuracy of the home address is even higher, considering that one of the strengths of this study was the utilization of the price per square meter of accommodations in the area of residence as a proxy for socioeconomic status. In fact, these data could be quite easy to collect on an ongoing basis and link to the socioeconomic status of the family. Moreover, this basic information could be of interest in order to target training programs for women living in difficult conditions, at a local level, with the ultimate aim of bridging the knowledge gap and addressing the obstacles that prevent or discourage a change in lifestyle or access to healthcare services.

## Abbreviations

ARO: adverse reproductive outcomes
CA: chromosomal abnormalities
CM: congenital malformations
CNS: central nervous system
HDD: hospital discharge data
LBW: low birth weight

## References

[1] Whitworth A, Aitken G, Anderson B, Ballas D, Dibben C, Heppenstall A, et al. Evaluations and improvements in small area estimation methodologies. Discussion Paper. National Centre for Research Methods. Sheffield, 2013.

[2] Riva M, Gauvin L, Barnett TA (2007). Toward the next generation of research into small area effects on health: a synthesis of multilevel investigations published since July 1998. J Epidemiol Community Health.

[3] Carstairs V, Elliott P, Wakefield JC, Best NG, Briggs DJ (2000). Socio-economic factors at area level and their relationship with health, in Spatial Epidemiology. Methods and Applications.

[4] Braubach M, Fairburn J (2010). Social inequities in environmental risks associated with housing and residential location—a review of evidence. Eur J Public Health.

[5] Brender JD, Maantay JA, Chakraborty J (2011). Residential proximity to environmental hazards and adverse health outcomes. Am J Public Health.

[6] Pirastu R, Pasetto R, Zona A, Ancona C, Iavarone I, Martuzzi M, et al. The health profile of population living in contaminated sites: SENTIERI approach. J Environ Pub Health. 2013. 10.1155/2013/939267 PMC3703355 23853611

[7] Coorevits P, Sundgren M, Klein GO, Bahr A, Claerhout B, Daniel C (2013). Electronic health records: new opportunities for clinical research. J Intern Med.

[8] Iezzoni LI (1997). Assessing quality using administrative data. Ann Intern Med.

[9] Shaban-Nejad A, Okhmatovskaia A, Izadi MT, Naderi N, Mondor L, Jauvin C (2013). PHIO: A Knowledge Base for Interpretation and Calculation of Public Health Indicators. Stud Health Technol Inform.

[10] Guerriero L, Ferdeghini EM, Viola SR, Porro I, Testi A, Bedini R (2011). Telematic integration of health data: a practicable contribution. Inform Health Soc Care.

[11] Buckingham WR (2012). The potential and pitfalls of geocoding electronic health records. WMJ.

[12] Do D, Finch B, Basurto-Davila R, Bird CE, Escarce J, Lurie N (2008). Does place explain racial health disparities? Quantifying the contribution of residential context to the black/white health gap in the United States. Soc Sci Med.

[13] Vrijheid M, Dolk H, Stone D, Abramsky L, Alberman E, Scott JE (2000). Socioeconomic inequalities in risk of congenital anomaly. Arch Dis Child.

[14] Miranda ML, Messer LC, Kroeger GL (2012). Associations between the quality of the residential built environment and pregnancy outcomes among women in North Carolina. Environ Health Perspect.

[15] Nieuwenhuijsen MJ, Dadvand P, Grellier J, Martinez D, Vrijheid M (2013). Environmental risk factors of pregnancy outcomes: a summary of recent meta-analyses of epidemiological studies. Environ Health.

[16] Dadvand P, Wright J, Martinez D, Basagaña X, McEachan RR, Cirach M (2014). Inequality, green spaces, and pregnant women: roles of ethnicity and individual and neighbourhood socioeconomic status. Environ Int.

[17] Timmermans S, Bonsel GJ, Steegers-Theunissen RP, Mackenbach JP, Steyerberg EW, Raat H (2011). Individual accumulation of heterogeneous risks explains perinatal inequalities within deprived neighbourhoods. Eur J Epidem.

[18] Vos AA, Posthumus AG, Bonsel GJ, Steegers EA, Denktaş S (2014). Deprived neighborhoods and adverse perinatal outcome: a systematic review and meta-analysis. Acta Obstet Gynecol Scand.

[19] Garcia-Subirats I, Perez G, Rodriguez-Sanz M, Ruiz-Muñoz D, Salvador J (2012). Neighborhood inequalities in adverse pregnancy outcomes in an urban setting in Spain: a multilevel approach. J Urban Health.

[20] Prospero E, Barbadoro P, Filippetti F, Appignanesi R, Ciavattini A, Carle F (2008). Occurrence of neural tube defects in pregnancy: an excess of cases in a 2773-km2 area in Central Italy. J Toxicol Environ Health A.

[21] EUROCAT. EUROCAT statistical monitoring protocol 2009. EUROCAT Central Registry, Univeristy of Ulster. 2011. http://www.eurocat-network.eu/accessprevalencedata/interpretationguide/calculationofprevalencerates. Accessed on 23 December 2015

[22] Cadum E, Costa G, Biggeri A, Martuzzi M (1999). Deprivazione e mortalità: un indice di deprivazione per l’analisi delle disuguaglianze su base geografica. Epidemiol Prev.

[23] Kulldorff M, Nagarwalla N (1995). Spatial disease clusters: Detection and Inference. Stat Med.

[24] Kulldorff M (1997). A spatial scan statistic. Communications in Statistics: Theory and Methods.

[25] Naus JI (1982). Approximations for Distributions of Scan Statistics. J Am Stat Assoc.

[26] Kulldorff M, Athas W, Feuer E, Miller BA, Key CR (1998). Evaluating cluster alarms: A space-time scan statistic and brain cancer in Los Alamos. Am J Public Health.

[27] Kulldorff M, Heffernan R, Hartman J, Assunção R, Mostashari F (2005). A space-time permutation scan statistic for disease outbreak detection. PLoS Med.

[28] Tango T (2007). A spatial scan statistic scanning only the regions with elevated risk. Advances in Disease Surveillance.

[29] Anselin L (1995). Local indicators of spatial association–LISA. Geogr Anal.

[30] Agenzia del Territorio. [Italian Revenue Agency. Official Italian Housing price statistics. http://www.agenziaentrate.gov.it/wps/content/nsilib/nsi/documentazione/omi/banche+dati/quotazioni+immobiliari. Accessed on 23 December 2015

[31] Croen LA, Shaw GM, Sanbonmatsu L, Selvin S, Buffler PA (1997). Maternal residential proximity to hazardous waste sites and risk for selected congenital malformations. Epidemiology.

[32] Marshall EG, Gensburg LJ, Deres DA, Geary NS, Cayo MR (1997). Maternal residential exposure to hazardous wastes and risk of central nervous sys-tem and musculoskeletal birth defects. Arch Environ Health.

[33] Vrijheid M, Dolk H, Armstrong B, Abramsky L, Bianchi F, Fazarinc I (2002). Chromosomal congenital anomalies and residence near hazardous waste landfill sites. Lancet.

[34] Diez Roux AV (2007). Neighborhoods and health: where are we and where do we go from here?. Rev Epidemiol Sante Publique.

[35] Renalds A, Smith TH, Hale PJ (2010). A systematic review of built environment and health. Fam Community Health.

[36] Gilligan C, Sanson-Fisher R, Eades C, D’Este C, Kay-lambkin F, Scheman S (2009). Identifying pregnant women at risk of poor birth outcomes. J Obstet Gyn.

[37] Räisänen S, Gissler M, Sankilampi U, Saari J, Kramer MR, Heinonen S (2013). Contribution of socioeconomic status to the risk of small for gestational age infants--a population-based study of 1,390,165 singleton live births in Finland. Int J Equity Health.

[38] Finlayson K, Downe S (2013). Why do women not use antenatal services in low-and middle-income countries? A meta-synthesis of qualitative studies. PLoS Med.

[39] Denny C, Floyd RL, Green PP, Hayes DK (2012). Racial and ethnic disparities in preconception risk factors and preconception care. J Womens Health (Larchmt).

[40] Larrañaga I, Santa-Marina L, Begiristain H, Machón M, Vrijheid M, Casas M (2012). Socio-Economic Inequalities in Health, Habits and Self-Care During Pregnancy in Spain. Matern Child Health J.

[41] Gavin AR, Nurius P, Logan-Greene P (2012). Mediators of adverse birth outcomes among socially disadvantaged woman. J Womens Health (Larchmt).

[42] Dwyer-Lindgren L, Gakidou E, Flaxman A, Wang H (2013). Error and bias in under-5 mortality estimates derived from birth histories with small sample sizes. Popul Health Metr.

[43] Lechat MF, Dolk H (1993). Registries of congenital anomalies: EUROCAT. Environ Health Perspect.

[44] Slama R, Ballester F, Casas M, Cordier S, Eggesbø M, Iniguez C (2014). Epidemiologic tools to study the influence of environmental factors on fecundity and pregnancy-related outcomes. Epidemiol Rev.

[45] Wang Y, Cross PK, Druschel CM (2010). Hospital discharge data: can it serve as the sole source of case ascertainment for population-based birth defects surveillance programs?. J Publ Health Manag Pract.

[46] Italian National Institute of Health (ISS). Integration of different health information systems for the analysis of frequencies of congenital malformation. (http://www.iss.it/binary/stat/cont/LavoriISS_Psn2014_2016.pdf). Accessed on 23 December 2015.

[47] Goodman M, Lakind JS, Fagliano JA, Lash TL, Wiemels JL, Winn DM (2014). Cancer cluster investigations: review of the past and proposals for the future. Int J Environ Res Public Health.

